# Measurement and interpretation of skin prick test results

**DOI:** 10.1186/s13601-016-0092-0

**Published:** 2016-02-23

**Authors:** J. P. M. van der Valk, R. Gerth van Wijk, E. Hoorn, L. Groenendijk, I. M. Groenendijk, N. W. de Jong

**Affiliations:** 1 Department of Internal Medicine, Section of Allergology, Erasmus MC, Burg. St. Jacobsplein 51, 3015CA Rotterdam, The Netherlands; 2 ICT Services, Erasmus MC, Rotterdam, The Netherlands

**Keywords:** Allergy, Cut-off value, Histamine equivalent index, Mean wheal diameter, Skin prick test

## Abstract

**Background:**

There are several methods to read skin prick test results in type-I allergy testing. A commonly used method is to characterize the wheal size by its ‘average diameter’. A more accurate method is to scan the area of the wheal to calculate the actual size. In both methods, skin prick test (SPT) results can be corrected for histamine-sensitivity of the skin by dividing the results of the allergic reaction by the histamine control. The objectives of this study are to compare different techniques of quantifying SPT results, to determine a cut-off value for a positive SPT for histamine equivalent prick -index (HEP) area, and to study the accuracy of predicting cashew nut reactions in double-blind placebo-controlled food challenge (DBPCFC) tests with the different SPT methods.

**Methods:**

Data of 172 children with cashew nut sensitisation were used for the analysis. All patients underwent a DBPCFC with cashew nut. Per patient, the average diameter and scanned area of the wheal size were recorded. In addition, the same data for the histamine-induced wheal were collected for each patient. The accuracy in predicting the outcome of the DBPCFC using four different SPT readings (i.e. average diameter, area, HEP-index diameter, HEP-index area) were compared in a Receiver-Operating Characteristic (ROC) plot.

**Results:**

Characterizing the wheal size by the average diameter method is inaccurate compared to scanning method. A wheal average diameter of 3 mm is generally considered as a positive SPT cut-off value and an equivalent HEP-index area cut-off value of 0.4 was calculated. The four SPT methods yielded a comparable area under the curve (AUC) of 0.84, 0.85, 0.83 and 0.83, respectively. The four methods showed comparable accuracy in predicting cashew nut reactions in a DBPCFC.

**Conclusions:**

The ‘scanned area method’ is theoretically more accurate in determining the wheal area than the ‘average diameter method’ and is recommended in academic research. A HEP-index area of 0.4 is determined as cut-off value for a positive SPT. However, in clinical practice, the ‘average diameter method’ is also useful, because this method provides similar accuracy in predicting cashew nut allergic reactions in the DBPCFC.

Trial registration: Trial number NTR3572

## Background

Standard diagnostics for Type-I acute allergic reactions to foods are based on the patient’s history combined with sensitisation tests and, optionally, a food challenge test [1]. Tests to measure sensitisation comprise in vitro specific IgE (sIgE) determination and skin prick testing (SPT). The outcome of the SPT can result in a variety of wheal shapes, and there are several methods to measure these outcomes. In clinical practice and in most academic research, it is common to characterize the wheal shape by the ‘average diameter’ [2]. However, with this method, it is implicitly assumed that the wheal may be described reasonably well by an ellipse or circle, which is not always the case in practice and this method is prone to errors [3]. For this reason, a more advanced scanning method for SPT measurement has been applied for more than a decade in the Erasmus Medical Centre in Rotterdam. To even further increase the accuracy of SPT results, the histamine-induced wheal size of the positive control might be considered as well to correct for skin histamine sensitivity. Furthermore, differences in technique of performing SPTs (inter-observer variability) contribute to the variation in wheal size [4]. We divided the area (or diameter) of the allergen-induced wheal by the area (or diameter) of the positive histamine-induced wheal controls to correct for these factors. This ratio is defined as the histamine equivalent prick (HEP)-index area (or diameter) or histamine-equivalent wheal sizes (HEWS) [5]. The first objective of this study is to compare different techniques of quantifying SPT results. The second objective is to determine a cut-off value for area, HEP-diameter and HEP-index area equivalent to the standard used average diameter cut-off value of 3 mm, whereby the HEP-index area is considered as the most important, because of the accuracy of this method (area measurement) and the correction for skin sensitivity (HEP-index measurement). The last objective is to study the accuracy of diagnosing cashew nut allergic reactions in the double-blind placebo-controlled (DBPCFC) tests with the 4 SPT methods.

## Methods

### Study design and patients’ characteristics

This study included a total of 172 children (trial number NTR3572). All patients underwent a SPT with cashew nut extract and a DBPCFC test with cashew nut. The mean age of the children was 8.8 years (range 2–17 years), with 102 boys (59 %) and 70 girls (41 %). Symptoms consistent with eczema were reported by 65 children (38 %), with asthma by 52 children (30 %) and with hay fever by 89 children (52 %). Medical ethical approval was obtained and all patients signed informed consent.

### Skin prick tests

The children underwent a SPT with homemade cashew nut extracts, a positive control (histamine 10 mg/ml ALK-Abello, Nieuwegein, The Netherlands) in duplicate and a negative control. Cashew nuts (roasted, unsalted) were homogenised mechanically, ground with a mortar, defatted by ether extraction, and subsequently the extracts were air-dried. A 10 % w/v extract in phosphate-buffered saline (PBS) with the pre-treated material was made and stored at −20 °C in small aliquots. Before testing the aliquots were defrosted and mixed. The SPT was performed by applying a drop of the allergen extract on the skin of the volar aspect of the forearm. Twenty minutes after the skin tests, the contours of the wheal were encircled with a fine-tip pen and transferred to a record sheet by translucent tape [6].

### Different techniques quantifying skin prick test results

**Fig. 1:**
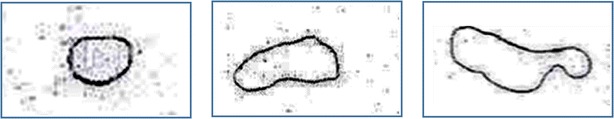
Typical observed wheal forms in SPT’s

**Fig. 2:**
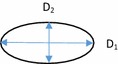
Definition of D1 and D2

The outcome of the SPT can result in a variety of wheal shapes, as shown in Fig. 1. To determine the average diameter, the mean value of the longest and the midpoint orthogonal diameter (mm) of the wheal were measured (Fig. 2). The area of the wheal was determined by using a flatbed scanner (Hewlett Packard) in combination with software earlier developed by Erasmus MC: Precise Automated Area Measurement of Skin Test (PAAMOST) [6, 7]. Mean values of two histamine-induced wheal sizes of the positive control were collected as well. Based on the measured data the HEP-indices were calculated for both the average diameter and area.

Consequently the four readings were defined as:

1. Average diameter (allergen-induced average wheal diameter).
2. Area (allergen-induced area measured by scanning device).
3. HEP-index diameter (allergen-induced average diameter divided by histamine-induced average diameter).
4. HEP-index area (allergen-induced area divided by the histamine-induced average area).

### Food challenge test

The children underwent a DBPCFC cashew nut test with an eight-step incremental dose regime. The children consumed 3180 mg cashew nut protein (22 cashew nuts) when the child consumed all 8 dose steps. The validated and standardised food challenge material used in the DBPCFC was prepared according to the recipe developed by Berber-Vlieg et al. [8]. The DBPCFC was considered as positive when (1) objective symptoms occurred, (2) when subjective symptoms occurred twice on three successive administration of the challenge material, or (3) when subjective symptoms persisted for more than 1 h [9]. In total, 137 children had a positive challenge test.

### Analysis

Receiver operating characteristics (ROC) curves and Area under the Curve (AUC) were calculated to evaluate the different SPT methods. An area under the curve of 0.9–1 is considered as excellent, 0.8–0.9 as good and 0.7–0.8 as fair [10]. All analyses were done with SPSS software, 20th edition.

## Results

### SPT

In total 172 SPT results with cashew, positive (in duplicate) -and negative control were evaluated. Median histamine wheal diameter was 5.38 mm (range 2.75–10.75 mm). All negative controls were negative. Mean variability between the duplicate measurements of histamine was 14 % (range 0–100 %). Median average diameter, area, HEP-diameter and HEP-index area of the SPT with cashew were 10.50 mm (range 0–26 mm), 71.8 mm^2^ (range 0–324.1 mm^2^), 1.83 (range 0–5.13) and 2.97 (range 0–15.16), respectively.

### Different techniques of interpreting skin prick test results

**Fig. 3:**
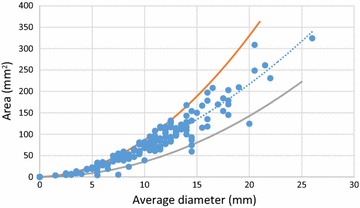
Average diameter (method 1) versus scanned area (method 2). A comparison is made between the common-used average diameter method and the scanned area method. Every *dot* represents one patient. The *dotted line* shows the trend line of the data. The lower bound value for α is 1 is shown by the *red line*. The upper bound value for α is 6.67 is shown by the *grey line*

As a first step of assessing the different techniques of interpreting the SPT results, a comparison is made between the common-used average diameter method (1) and the scanned area method (PAAMOST) (2). These two methods are compared in a scatterplot in Fig. 3. Every dot represents one patient. The dotted line shows the trend line of the data.

The average diameter on the horizontal axis in Fig. 3 is defined as the mean value of the longest (*D*_*1*_) and the midpoint orthogonal diameter (*D*_*2*_) of the wheal, as shown in Fig. 2:

1 $$D = \left( {\frac{{D_{1} + D_{2} }}{2}} \right)$$

In most studies, the average diameter is presented, while the originally measured values of D1 and D2 are not shown. This results in loss of crucial information. Without the parameters D1 and D2, there is no indication about the original shape of the wheal. To avoid this, we introduce, next to the parameter D, the parameter α as the ratio between *D*_*1*_ and *D*_*2*_:

2 $$\alpha = \frac{{D_{1} }}{{D_{2} }}$$

The combination of parameters D and α contains exactly the same information as the measured parameters D1 and D2. A value for α close to 1.0 indicates a circular shaped wheal, higher values indicate an elliptical shaped wheal.

In our study population of 172 patients, the parameter α varies between 1.0 and 6.67. Assuming that we can reasonably well estimate the wheal size by an ellipse, the area of the wheal (*A*) is defined as:

3 $$A = \frac{\pi }{4}D_{1} D_{2}$$

In Eq.( 3) the wheal area is defined as a function of *D*_*1*_ and *D*_*2*_, while the wheal size is commonly characterized by the average diameter, in particular in method 1. Combining Eqs. (1) to (3), the wheal area can be rewritten as a function of the average diameter *D* and the ratio α:

4 $$A = \frac{\alpha }{{\left( {1 + \alpha } \right)^{2} }}\pi D^{2}$$

The lower bound value of α is 1.0 (*D*_1_ = *D*_2_). In this case, the wheal shape is circular and Eq. (4) simplifies to the well-known formula describing the area of circle, *A* = *π/*4*·D* ^*2*^. In Fig. 3, this lower bound case (area as a circle) is shown by the red line. Based on our set of 172 patients, the upper bound value of α is 6.67. Substituting α = 6.67 into Eq. 4, the upper bound (area as an ellipse) is obtained. This is shown by the grey line in Fig. 3. Nearly all 172 dots are lying in between these two lines, with only a few exceptions. The reason for these outliers is that an ellipse could not sufficiently well represent the shape of these wheals. From Fig. 3 it can be concluded that characterizing the wheal size by the average diameter method could be rather inaccurate. For a given average wheal diameter, the actual wheal area could vary between 50 % under and 50 % above the trend line, visually in between the red and grey line. For example, if the mean wheal diameter is 15 mm, the real wheal area could lie between 80 mm^2^ (α = 6.67) and 176 mm^2^ (α = 1.0), which is a rather large variation. Figure 3 shows also that the absolute error grows with wheal size. This inaccuracy, of up to 50 %, is completely eliminated if one applies the scanning method, i.e. method 2.

If for practical reasons, one would like to use the average diameter method, the ‘best’ relationship between the average diameter *D* and the wheal area *A* may be obtained from the dotted trend line in Fig. 3. This line can be estimated by the following equation:

5 $$A = \frac{\pi }{6}D^{2}$$

It is interesting to note that this expression is rather different than the commonly used expression *A* = *π/*4*·D* ^*2*^, which implicitly assumes a circular wheal shape.

**Fig. 4:**
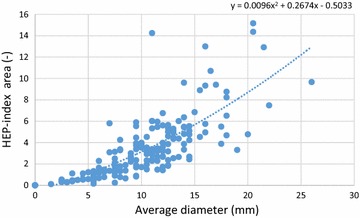
Average diameter (method 1) versus HEP-index area (method 4)

To determine the cut-off value for HEP-index area equivalent to the standard used 3 mm average diameter cut-off value [11], comparison is made between the average diameter method (1) and the scanned HEP- area method (4). These two methods are compared in a scatterplot in Fig. 4. The dotted line shows the trend line of the data. This trend line can be estimated by the following equation:

6 $$HEP - index \;area = 0.0096 D^{2} + 0.2674 D - 0.5033$$

Substituting *D* = 3 mm into Eq. (6), the HEP-index area is obtained and results in 0.4. Therefore, a HEP-index area value of 0.4 is considered as the cut-off value for a positive SPT.

The cut-off values for area and HEP-index diameter were measured on the same method. This results in an area and HEP-index diameter cut-off values of 4.71 mm^2^ and 0.6, respectively.

### Accuracy of diagnosing cashew allergy

**Fig. 5:**
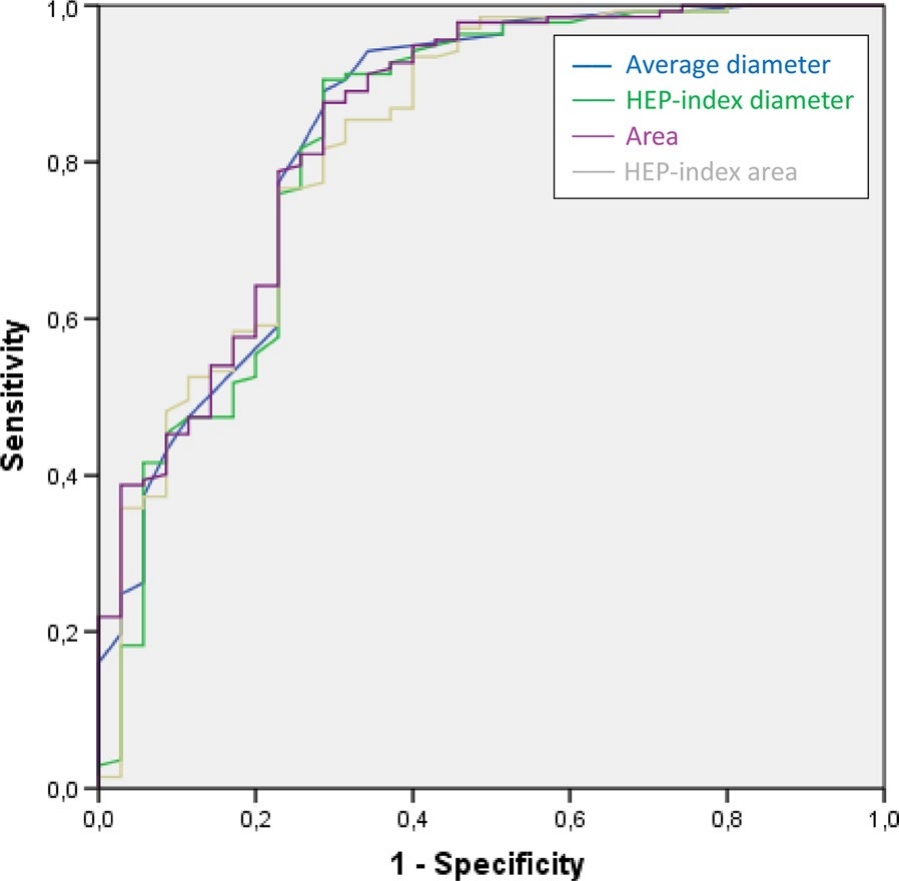
Receiver-operating characteristic* curves* for the 4 SPT methods

To study the accuracy of diagnosing cashew nut allergy with the four SPT methods, a ROC plot was generated. The four SPT methods, i.e. the average diameter, area, HEP-index diameter and HEP-index area, yielded a comparable area under the curve of 0.84, 0.85, 0.83 and 0.83, respectively. All four SPT methods were considered as good and equally accurate in diagnosing cashew nut allergy (Fig. 5).

## Discussion

To determine the outcome of the SPT, it is common to characterize the wheal shape by the ‘average diameter’. However, this method is prone to errors, because it is assumed that the wheal size varies between a circle and an ellipse. In fact, the wheals have pseudopodia and interpretation based on two orthogonal diameters is not accurate. This study showed that for a given average wheal diameter, the actual wheal area could vary quite significantly and this inaccuracy grows with wheal size. This inaccuracy is completely eliminated if one applies the scanning method. This more precise method for measuring the wheal size area is previously described by Pijnenborg et al. [12]. The scanning method is also fast, easy in use, has a high reproducibility and is very useful in scientific research [2, 6, 12, 13].

To even further increase the accuracy of SPT results, the HEP-index can be calculated, to rule out differences in skin reactivity. There are several factors that contribute to this difference, e.g., poly-sensitised patients and patients with mould sensitisation have significantly higher skin reactions [14] and the skin response varies in different ethnicities [15]. Furthermore, differences in technique of performing SPTs (inter-observer variability) contribute to the variation in wheal size [4]. To correct for these factors, the calculation of the HEP-index is useful and also easy to determine with the scanning method.

Notwithstanding all advances of the scanning method inclusive the HEP-index calculation, the ‘average diameter’ method is as accurate in diagnosing cashew nut allergy as the ‘HEP-index area’ method. Therefore, the ‘average diameter’ method can be used if there is no scanning device available. However, the ‘best’ relationship between the average diameter and the wheal area can be better estimated by the equation $A = \frac{\pi }{6}D^{2}$ instead of the equation $A = \frac{\pi }{4}{\text{D}}^{2}$. Therefore, if one wishes to calculate the area out of the average diameter for e.g. research purposes, the equation $A = \frac{\pi }{6}D^{2}$ should be used to approximate the area most accurate.

## Conclusions

This study demonstrates that the scanning method for SPT measurement is more accurate to measure the wheal area in a Type-I allergy than the average diameter. The average wheal diameter gives an overestimation or underestimation of the actual area up to 50 %. It is possible to correct for skin sensitivity and inter-observer variability by using the ‘HEP-index area’ method. The HEP-index area value 0.4 can be considered as an equal cut-off value of 3 mm wheal average diameter. However, in clinical practice, the ‘average diameter method’ is also useful, because this method is equally accurate in predicting cashew nut allergic reactions in the DBPCFC tests.

## Abbreviations

AUC: Area under the curve
DBPCFC: Double-blind, placebo-controlled food challenge
HEP: Histamine equivalent prick
sIgE: Specific IgE
ROC: Receiver-operating characteristic
SPT: Skin prick test
